# Serum resistin: A possible link between inflammation, hypertension and coronary artery disease

**DOI:** 10.12669/pjms.35.3.274

**Published:** 2019

**Authors:** Sobia Niaz, Javaria Latif, Shaista Hussain

**Affiliations:** 1 *Sobia Niaz, Assistant Professor, Department of Physiology, Fatima Memorial Hospital College of Medicine & Dentistry, Lahore, Pakistan*; 2 *Javaria Latif, Assistant Professor, Department of Physiology, Amna Inayat Medical College, Lahore, Pakistan*; 3 *Shaista Hussain, Assistant Professor, Department of Physiology, Rahber Medical College, Lahore, Pakistan*

**Keywords:** Resistin, hypertension, coronary artery disease, inflammation, C-reactive protein, Total leucocyte count

## Abstract

**Background & Objectives:**

Inflammation is considered as the main triggering factor in evolution of atherosclerotic pathology of heart and blood vessels. Resistin, an inflammatory cytokine is proved to be a main mediator of initiation and progression of mechanisms leading to atherosclerosis, hypertension and ultimately to coronary artery disease. Our objective was to compare the levels of serum resistin, C-reactive protein and total leucocyte count in subjects of hypertension and coronary artery disease; and to observe the correlation of serum resistin with CRP and TLC in the study participants.

**Methods:**

Eighty selected participants were divided into four equal groups including normal healthy participants, newly diagnosed cases of hypertension, stable angina pectoris and myocardial infarction, both with hypertension. The study was conducted in the physiology department of Post Graduate Medical Institute Lahore, during 2013. After consent, history and examination, fasting blood samples of the participants were collected. Serum resistin and C-reactive protein were determined by using standard techniques of enzyme linked immunosorbent assay, while total leukocyte count by automated hematology analyzer.

**Results:**

The values of serum resistin, C- reactive protein and total leukocyte count were found significantly raised in patients of hypertension, angina pectoris and myocardial infarction with hypertension as compared to normal participants (p<0.001 for all). Significantly positive correlation of resistin was observed with TLC only in hypertensive patients of myocardial infarction (r = 0.459, n = 20, p = 0.042) while in other study groups correlation between resistin and TLC as well as CRP was non-significant.

**Conclusion:**

Serum resistin levels along with CRP and TLC are significantly raised in patients of hypertension and coronary artery disease while resistin levels revealed significantly positive correlation with TLC in hypertensive patients of myocardial infarction.

## INTRODUCTION

Resistin, an adipocytokine, was initially discovered in a research conducted on obese rodents to probe the missing link between obesity, diabetes mellitus and insulin resistance. Later it was proved to be a member of the family of resistin-like molecules (RELMs).^1^ These molecules also termed as “FIZZ” (found in inflammatory zone); include resistin/FIZZ3, RELMα/FIZZ1 and RELMβ/FIZZ2 which are widely distributed in different body tissues.^2^

In humans, resistin is mainly secreted by bone marrow, monocytes and macrophages. Its production from theses blood cells is found to be strongly related to endotoxemia.^3^ Resistin mediates its inflammatory role by intracellular activation of nuclear transcription factor-kappa B which leads to secretion of various cytokines. These cytokines initiate multiple intracellular inflammatory cascades, promoting endothelial dysfunction, vascular smooth muscle cell (VSMC) proliferation and migration leading to atherothrombosis, hypertension and coronary artery disease (CAD).^4^

Hypertension is a clinical condition which effects heart and blood vessels due to persistent elevation of blood pressure (BP). If untreated, hypertension can lead to various morbidities such as CAD, heart failure, stroke, renal failure and eventually early death.^5^

Angina pectoris (AP) and myocardial infarction (MI) are clinical presentations of CAD produced either by transient or prolonged myocardial ischemia respectively.^6^ World health organization ranked CAD as one of the leading disease causing mortality and morbidity in developed and developing countries. In Pakistan, considered as the second most fatal disease, it is responsible for 11% of adult deaths.^7^

C-reactive protein (CRP), member of pentraxin family, is a nonspecific marker having amplified secretions in variou inflammatory conditions. Tissue inflammation leads to release of IL-6 and TNF-α which activates the hepatic production of CRP augmenting further release of cytokines.^8^

Leukocytes are defensive blood cells rendering immunity to our body. Total leucocyte count (TLC), if raised, indicates inflammation in the body so it is widely used as a reliable diagnostic and prognostic test in multiple researches.^9^

It is a well known fact that coronary artery disease is a consequence of low grade vascular inflammation. Expression of resistin from inflammatory blood cells augmenting release of cytokines and causing sub-endothelial damage reveals its association with subclinical atherosclerosis resulting in hypertension and CAD. So this study is designed to determine the possible inflammatory role of resistin by observing its association with other documented inflammatory biomarkers such as TLC & CRP in cases of variable degrees of cardiovascular disease i.e. hypertension, angina pectoris and myocardial infarction. Our findings have suggested that serum resistin, CRP and TLC are significantly raised in study groups as compared to healthy participants. Among these groups, resistin is positively correlated with TLC only in hypertensive patients of MI.

## METHODS

This comparative study was carried out in the physiology department of Post Graduate Medical Institute Lahore, in cooperation with the Punjab Institute of Cardiology Lahore. After approval of the Advanced Science and Research Board of the University of Health Sciences Lahore, it was completed in almost one year i.e. during 2013.

Sampling was done by non-probability convenience technique to select eighty participants between 30-55 years of age and divided into four groups. Sample size was calculated by using the following formula:


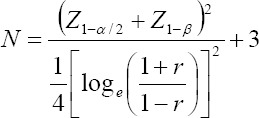


In this formula “N” is the sample size, “Z _1 – β_” is the desired power of study (95%), “Z _1-α/2_” is the desired level of significance (5%) and “r” is the correlation coefficient.

Each group consisted of 20 participants with equal number of males (10) and females (10). Groups were categorized as Group A, including healthy normotensives (BP equal to or less than 120/80 mm Hg); group B comprising of newly diagnosed cases of hypertension, group C consisting of newly diagnosed cases of stable angina pectoris with hypertension and group D with cases of acute myocardial infarction with hypertension.

Newly diagnosed case is the patient who has been diagnosed for the first time within last thirty days or one month. Patients with hypertension, angina pectoris or myocardial infarction were excluded from group A. While smokers, subjects with obesity (BMI equal to or more than 30 kg/m^2^)^10^, diabetes mellitus, any acute or chronic inflammation, surgical or cardiac intervention, congenital or valvular heart disease were excluded from all groups.

After taking written informed consent, detailed history of the participants was inquired. Blood pressure was estimated by using standard technique with mercury sphygmomanometer. Any participant with blood pressure equal to or more than 140/90 mm Hg was taken as hypertensive.^5^

By using aseptic technique, 5 ml of fasting blood sample was drawn with a disposable syringe. Of the collected blood, 3 ml was placed in the serum vial for estimation of serum resistin and CRP while 2 ml in CBC vial for TLC. After centrifugation at a speed of 5000 rev/min for 10 minutes, serum was drawn and stored in aliquots at -20°C till further analysis. Total leucocyte count was estimated on the same day by automated hematology analyzer, Sysmex KX-21 (Sysmex Corporation, Kobe, Japan).

Resistin was determined by sandwich ELISA technique using kit of Creative Diagnostics (USA) while C-reactive protein was estimated by high sensitivity enzyme immunoassay test (Biocheck, Inc. Foster City, CA); both with automated analyzer, Stat Fax 303 of Awareness Technology (USA).

Data analysis was performed by using SPSS version 17.0. Normality of data was assessed by Shapiro-Wilk test. Normally distributed data were presented as mean ± SD (standard deviation) and analyzed by One Way ANOVA for statistical significance along with Post-hoc Tukey’s test for comparison between the groups. Non-normally distributed data were presented as median with IQR (interquartile ratio) and analyzed by Kruskal Wallis test followed by Dunn-Bonferroni test for post hoc group wise comparison. Correlation between serum resistin and TLC was observed by applying Pearson’s correlation coefficient, while Spearman’s rank test for the correlation between resistin & CRP. Statistically, p-value ≤ 0.05 was considered as significant.

## RESULTS

The study included 80 participants equally divided in four groups. Values of serum resistin, C-reactive protein and total leucocyte count are presented in Table-I. Statistically significant increase in the levels of resistin, CRP and TLC was seen in patients of hypertension (group B), stable angina pectoris with hypertension (group C) and myocardial infarction with hypertension (group D) as compared to normal subjects of group A (p < 0.001 for all). Table-II summarizes the comparison of these three parameters by Post hoc analysis.

**Table-I:** Comparison of serum resistin, CRP and TLC values in groups A, B, C and D.

Parameter	Group A n = 20	Group B n = 20	Group C n = 20	Group D n = 20	p – value

	mean ± SD/ Median (IQR)	mean ± SD/ Median (IQR)	mean ± SD/ Median (IQR)	mean ± SD/ Median (IQR)	
Resistin (ng/ml)	6.80 ± 1.01	16.73 ± 3.78	17.51 ± 8.04	21.07 ± 7.12	<0.001*
CRP (mg/L)	0.50 (0.50-0.95)	4.00 (2.60-10.12)	18.20 (11.85-35.60)	42.10 (26.30-48.50)	<0.001*
TLC (×10^3^/uL)	6.13 ± 0.84	7.41 ± 1.36	8.79 ± 1.32	13.17 ± 3.22	<0.001*

Group A = Normal participants Group B = Hypertensive patients Group C = Patients of angina pectoris with hypertension Group D = Patients of myocardial infarction with hypertension n = number of subjects Values are given as mean ± SD for normally distributed variables and median (IQR) for non-normally distributed variables

* = significant

**Table-II:** Post hoc comparison of serum resistin, CRP and TLC levels between groups A, B, C and D.

Parameter	Group A Vs Group B	Group A Vs Group C	Group A Vs Group D	Group B Vs Group C	Group B Vs Group D	Group C Vs Group D
Serum Resistin	<0.001*^a^	<0.001*^a^	<0.001*^a^	0.973^a^	0.086^a^	0.208^a^
Serum CRP	0.041*^b^	<0.001*^b^	<0.001*^b^	0.042*^b^	<0.001*^b^	0.299^b^
TLC	0.162^a^	<0.001*^a^	<0.001*^a^	0.113^a^	<0.001*^a^	<0.001*^a^

Group A = Normal participants Group B = Hypertensive patients Group C = Patients of angina pectoris with hypertension Group D = Patients of myocardial infarction with hypertension

a p-value is generated by Post hoc Tukey’s Test,

b p-value is generated by Dunn-Bonferroni Test

* = significant

Statistically significant positive correlation was observed between serum resistin and TLC only in group D i.e. patients of myocardial infarction with hypertension (r=0.459, n=20, p=0.042) while correlation was non-significant between serum resistin and CRP in all four study groups (Table-III).

**Table III:** Correlation between serum resistin, CRP and TLC in groups A, B, C and D.

Parameter	Group A		Group B		Group C		Group D	

	r	p	r	p	r	p	R	p
Resistin and CRP	0.266^a^	0.257^a^	-0.407^a^	0.075^a^	-0.066^a^	0.782^a^	-0.352^a^	0.128^a^
Resistin and TLC	0.343^b^	0.139^b^	0.067^b^	0.778^b^	0.298^b^	0.203^b^	0.459^b^	0.042*^b^

Group A = Normal participants Group B = Hypertensive patients Group C = Patients of angina pectoris with hypertension Group D = Patients of myocardial infarction with hypertension r = correlation coefficient

a Analysis of correlation by Spearman’s rank test,

b Analysis of correlation by Pearson’s correlation coefficient,

* = significant

## DISCUSSION

Hypertension and coronary artery disease, both are known to occur as a sequel of low grade inflammatory process in the vascular endothelium i.e. atherosclerosis. Progression of the pathological changes in diameter and flow of circulatory passages ultimately lead to narrowing and occlusion of the arteries.^5^ Resistin is mainly expressed from inflammatory white blood cells and is thought to participate in the release of a wide array of cytokines revealing its possible involvement in inflammation.^3^ This inflammatory link was explored in this study by observing the serum levels of resistin, CRP and TLC in normal subjects and in patients with variable severity of cardiovascular disease.

Our results demonstrated progressive increase in serum resistin levels in patients of hypertension and coronary artery disease as compared to normal subjects. These results are concurrent with the results of an analytical research which revealed raised resistin levels in hypertensive patients as compared to normotensives. Resistin is thought to play this role by altering the renin angiotensin pathway and vascular remodeling.^11^ In another study, strong association of raised resistin levels with increased future risk of developing hypertension was observed.^12^

Different cohort researches derived similar relationship of resistin with myocardial inflammation when strong association of raised serum resistin levels was observed in Spanish adults with the increased future risk of CAD^13^ as well as with the severity and outcome of cardiovascular disease in a multiethnic research. But the exact role of resistin in causation of cardiac inflammation was not explored.^14^

Serum C-reactive protein is a well known inflammatory marker. Various studies have proved its proatherogenic role in vascular inflammation leading to atherosclerosis and CAD.^8^ In this research, we have observed the parallel increase of serum resistin and CRP with increasing pathogenesis of the disease. Similar results were obtained in a research conducted on patients of MI, stable and unstable AP; in which progressive rise in serum resistin levels and CRP was noted with increasing myocardial inflammation.^15^ Significant positive correlation of resistin with CRP was observed in hypertensive patients with peripheral arterial disease^16^ as well as in female nurses with endothelial dysfunction.^17^ This link of resistin with CRP and inflammation was observed in other diseases also such as in patients of dementia due to neurovascular inflammation^18^ and in patients of chronic obstructive pulmonary disease. The increase in serum levels of resistin and other inflammatory markers was parallel to the inflammatory load of the disease.^19^

Elevated total leucocyte count indicates any acute or chronic inflammation in the body. Resistin is related to leucocyte count as its major expression is by the inflammatory blood cells.^9^ The present study demonstrated increase in serum resistin and TLC in all study groups as compared to normal subjects. Moreover significant positive correlation of resistin was observed with TLC in hypertensive patients of myocardial infarction. Comparable results were obtained in a research conducted in Karachi on patients of myocardial infarction, stable and unstable angina pectoris.^20^ Connection of raised resistin levels with inflammatory biomarkers like hsCRP and TLC was also established in a study conducted to observe the association of carotid artery intimal thickness with the myocardial impairment^21^ and in patients with mild acute pancreatitis showing its relevance to extent of inflammation as well as outcome of the disease.^22^

Despite lot of supporting data, we came across with controversial results also. In a comparative study on smokers and non smokers, no correlation was found between serum resistin and CRP. It was thought to be due to inclusion of young healthy subjects.^23^ Similarly, in patients of rheumatoid arthritis, serum resistin levels were not associated with CRP and TLC. It was explained on the basis of the fact that the inflammatory markers raise much in their synovial fluid as compared to serum.^24^

The present study supports the inflammatory role of resistin in patients of hypertension and coronary artery disease. Future endeavors in this direction, overcoming the study limitations, will help in better understanding of the links between the triad of resistin, inflammation and coronary artery disease.

### Limitations of Study

Sample size was insufficient for generalization of the results. Moreover association of resistin with certain confounding and risk factors of CAD was not observed. This limited our exploration regarding cause and effect relationship of resistin with inflammation.

## CONCLUSIONS

Serum levels of resistin, C-reactive protein and total leucocyte count are significantly raised with increasing inflammatory pathogenesis of the cardiovascular disease in patients of hypertension and coronary artery disease as compared to normal subjects. As circulating resistin levels are associated with inflammatory markers, it appears that by inducing and propagating inflammatory processes especially in heart and blood vessels, resistin leads to the pathogenesis of hypertension and coronary artery disease.

### Authors’ Contribution

**SN:** Principle investigator/researcher of the project, conception and design of study and data acquisition.

**JL:** Data analysis, interpretation of results and critical review of the article.

**SH:** Article drafting and final version of the Manuscript.
